# Facial nerve compression by the posterior inferior cerebellar artery causing facial pain and swelling: a case report

**DOI:** 10.1186/1752-1947-8-105

**Published:** 2014-03-25

**Authors:** Rebecca L Batten, Wan-Fai Ng

**Affiliations:** 1 Clinical Research Facility, Level 6, Leazes Wing, Royal Victoria Infirmary, Newcastle upon Tyne NE1 4PE, UK; 2 Institute of Cellular Medicine, Medical School, Framlington Place, Newcastle University, 4th Floor, William Leech Building, Newcastle upon Tyne NE2 4HH, UK

**Keywords:** Facial pain, Hemifacial spasm, Facial pain, Vascular compression, Posterior inferior cerebellar artery, Vascular compression

## Abstract

**Introduction:**

We report an unusual case of facial pain and swelling caused by compression of the facial and vestibulocochlear cranial nerves due to the tortuous course of a branch of the posterior inferior cerebellar artery. Although anterior inferior cerebellar artery compression has been well documented in the literature, compression caused by the posterior inferior cerebellar artery is rare. This case provided a diagnostic dilemma, requiring expertise from a number of specialties, and proved to be a learning point to clinicians from a variety of backgrounds. We describe the case in detail and discuss the differential diagnoses.

**Case presentation:**

A 57-year-old Caucasian woman with a background of mild connective tissue disease presented to our rheumatologist with intermittent left-sided facial pain and swelling, accompanied by hearing loss in her left ear. An autoimmune screen was negative and a Schirmer’s test was normal. Her erythrocyte sedimentation rate was 6mm/h (normal range: 1 to 20mm/h) and her immunoglobulin G and A levels were mildly elevated. A vascular loop protocol magnetic resonance imaging scan showed a loop of her posterior inferior cerebellar artery taking a long course around the seventh and eighth cranial nerves into the meatus and back, resulting in compression of her seventh and eighth cranial nerves. Our patient underwent microvascular decompression, after which her symptoms completely resolved.

**Conclusion:**

Hemifacial spasm is characterized by unilateral clonic twitching, although our patient presented with more unusual symptoms of pain and swelling. Onset of symptoms is mostly in middle age and women are more commonly affected. Differential diagnoses include trigeminal neuralgia, temporomandibular joint dysfunction, salivary gland pathology and migrainous headache. Botulinum toxin injection is recognized as an effective treatment option for primary hemifacial spasm. Microvascular decompression is a relatively safe procedure with a high success rate. Although a rare pathology, posterior inferior cerebellar artery compression causing facial pain, swelling and hearing loss should be considered as a differential diagnosis in similar cases.

## Introduction

Hemifacial spasm (HFS) due to vascular loop compression of the seventh cranial nerve at the root exit zone at the cerebellopontine angle has been well documented [1]. HFS is characterized by unilateral clonic twitching, initially affecting the orbicularis oculi muscle then progressing to the paranasal and perioral muscles [2]. It is the result of erratic nerve conduction and hyperstimulation of the facial nerve nucleus [3].

Onset of symptoms is between 30 and 70 years of age, mostly in middle age, and women are more commonly affected. One study reported that 72% of patients with HFS were female. Out of 74 patients, 17.5% reported concurrent auditory and vestibular symptoms [4]. Wang and Jankovic found that the left side was affected in 56% of patients, and bilateral HFS was rare [5].

Occasionally, patients exhibit symptoms of trigeminal neuralgia, HFS and glossopharyngeal involvement. This is recognized as combined hyperactive dysfunction syndrome, and affected 2.8% of patients in one case review [6]. Patients with this combination tend to be older (mean age 66.6 years), again with a female predominance. Aging and hypertension accelerate the progressive arteriosclerotic vasculoarchitechtural changes that occur within the vertebrobasilar system [6].

## Case presentation

A 57-year-old woman presented in 1995 with a six-week history of acute onset polyarthralgia. She also had Raynaud’s phenomenon, dry eyes, dyspepsia, intermittent night sweats, brittle nails, intermittent hair loss, mouth ulcers and fatigue. A provisional diagnosis of mild connective tissue disease (Sjögren’s or lupus) was made, based on her symptoms and a single positive doubled-stranded deoxyribonucleic acid (DNA) result.

Her past medical history included Hashimoto’s thyroiditis and osteoarthritis. In 2007, she was infected with a sheep bot fly while in Morocco. She subsequently had larvae removed from her left maxillary sinus and her right eye by our ear, nose and throat team.

In 2010, she re-presented to our rheumatology department with intermittent left-sided facial pain and swelling, accompanied by hearing loss in her left ear. She also described gradually worsening epiphora, blepharospasm and left eye swelling.

An autoimmune screen was negative, including anti-Ro and anti-La, anti-neutrophil cytoplasmic antibodies, autoantibody and antinuclear antibodies. Her erythrocyte sedimentation rate was 6mm/h (normal range: 1 to 20mm/h) and her levels of immunoglobulin G and A were mildly elevated. A Schirmer’s test was also normal. In view of these results, it was felt that her symptoms were unlikely to be due to connective tissue disease.

Magnetic resonance imaging (MRI) of her sinuses, skull base and parotid glands showed only mild thickening of her sinuses. She was referred to our ear, nose and throat team for further investigation. A tympanogram demonstrated low tone loss and negative pressure. An ultrasound scan of the left side of her face found no cause for the swelling, and a flexible naso-endoscopy was normal.

In 2012, our patient was referred to our ophthalmology team because of the persisting symptoms. She had been started on carbamazepine and botulinum toxin injections to her facial muscles, with minimal symptomatic improvement. A second MRI scan was performed and reviewed by a neuroradiologist. This scan suggested vascular compression of her left facial nerve. A vascular loop protocol MRI showed a loop of her posterior inferior cerebellar artery taking a long course around the seventh and eighth cranial nerves into the meatus and back, resulting in compression of her seventh and eighth cranial nerves.

Our patient was referred to our neurosurgical team for microvascular decompression. Her symptoms of pain, epiphora, blepharospasm and swelling completely resolved following this procedure.

## Discussion

Compression of the seventh and eighth nerves characteristically results in HFS and vestibular and auditory symptoms. Our patient presented with facial swelling and pain as her main symptoms. To the best of our knowledge, swelling associated with HFS has been documented in the literature only once: this case report suggested that the underlying mechanism was persistent clonic contractions hindering venous and lymphatic return, thus causing persistent swelling [7].

Facial pain due to trigeminal nerve vascular compression has been well documented, however the imaging in our patient did not identify any trigeminal nerve pathology, despite its anatomical proximity to the seventh nerve root exit zone. It is possible that the pain our patient was describing was geniculate neuralgia - a result of compression of the nervus intermedius, which travels between the seventh and eighth cranial nerves.

Secondary causes of HFS include tumor, trauma, demyelinating lesions, arteriovenous malformation and aneurysm at the cerebellopontine angle. However, a secondary cause is rare, occurring in only 0.5% of patients in one large (1,642 patients) case review [8].

The differential diagnosis to be considered in patients who present with facial pain and swelling is wide-ranging. Temporomandibular joint dysfunction is commonly associated with rheumatic disease. Clinical features include restricted maximal mouth opening, locking or dislocation, crepitation, masticatory muscle tenderness, and restricted laterotrusion [9].

Sensory trigeminal neuropathy presents with unilateral or bilateral facial numbness, paresthesia, dysesthesia, or pain, with a slowly progressive course. It has been recognized as a presenting feature of mixed connective tissue disease [10], systemic lupus erythematosus and Sjögren’s syndrome [11].

Salivary gland pathology due to hyposalivation is a recognized feature of connective tissue disease, and includes salivary gland infection, parotid necrosis, submandibular lymphadenopathy and primary salivary gland lymphoma. Focal sialadenitis due to Sjögren’s syndrome (primary or secondary) presents with increased oral caries, oral dryness, candida infection, difficulty tolerating oral prostheses and facial pain. It is recognized in up to 90% of patients with mixed connective tissue disease [11].

Migrainous headache has also been well documented in a number of connective tissue diseases, including systemic lupus erythematosus and Sjögren’s syndrome [11].

Our patient had an unusual history of sheep bot fly infection of the sinuses, which complicated the investigation and diagnosis, but was an important differential to exclude as a cause of her facial pain.

Imaging of the posterior fossa structures is ideally performed under MRI vascular loop protocol. One study reviewed 83 patients with evidence of root exit zone seventh cranial nerve compression on MRI, and found that only 16% were symptomatic [12]. This suggests that intervention should be guided by symptoms rather than radiologic findings alone. Another study reviewed MRI of 74 patients with symptoms of HFS and found that imaging findings had no impact on patient management, as most had symptom resolution with botulinum A toxin treatment. This suggests that where resources are limited, imaging may be reserved for patients with red flag or non-resolving symptoms [4].

Botulinum toxin injection is recognized as an effective treatment option for primary HFS, with an excellent safety and tolerability profile [13]; however, our patient did not receive any benefit from this treatment. The evidence for the role of botulinum toxin in secondary HFS is limited due to the rarity of the condition. A recent case report suggests that it is an effective treatment option in secondary HFS [14].

Hyun *et al.* performed a prospective study of microvascular decompression of HFS in 1,174 operations. Of these, 94.1% of patients were cured of their symptoms and 5.9% had residual spasm. Complications included transient hearing loss (2.6%), permanent hearing loss (1.1%), transient facial weakness (7.6%), permanent facial weakness (0.7%), cerebrospinal fluid leakage (0.25%), and cerebellar hemorrhage or infarction (0.17%). There were no operative deaths [15]. Overall, this is a relatively safe procedure with a high success rate.

## Conclusion

We highlight the complexity of diagnosing facial pain in a patient with connective tissue disease, caused by the variety of orofacial manifestations of disease. In this case it was the role of the rheumatologist to exclude an underlying inflammatory cause of our patient’s pain prior to referring her for further investigation. Primary and secondary HFS are rare but important causes of facial pain to consider, because surgical intervention is generally safe with good outcomes.

## Consent

Written informed consent was obtained from the patient for publication of this case report. A copy of the written consent is available for review by the Editor-in-Chief of this journal.

## Abbreviations

HFS: Hemifacial spasm; MRI: Magnetic resonance imaging.

## Competing interests

The authors declare that they have no competing interests.

## Authors’ contributions

WFN identified the case report and followed up our patient in clinic. RB reviewed the case notes and was a major contributor in writing the manuscript. Both authors read and approved the final manuscript.
